# Peri-Injury Stearidonic Acid Ethanolamide Modulates Astroglial Reactivity-Associated Markers in a Mouse Model of Mild Traumatic Brain Injury

**DOI:** 10.3390/pathophysiology33040078

**Published:** 2026-10-20

**Authors:** Anastasia Girich, Igor Manzhulo, Darya Ivashkevich, Arina Ponomarenko, Anna Tyrtyshnaia

**Affiliations:** A.V. Zhirmunsky National Scientific Center of Marine Biology, Far Eastern Branch, Russian Academy of Sciences, Vladivostok 690041, Russia; egoraeva_a@bk.ru (A.G.); i-manzhulo@bk.ru (I.M.); owncean@yandex.ru (D.I.); arina.ponomarenko.93@mail.ru (A.P.)

**Keywords:** N-acylethanolamines, stearidonic acid ethanolamide, mild traumatic brain injury, astroglia, neuroinflammation, PPAR-α, PPAR-γ, neurotrophic factors

## Abstract

Background/Objectives: Mild traumatic brain injury (mTBI) can cause persistent neuroinflammation and astroglial reactivity. This study examined whether peri-injury administration of stearidonic acid ethanolamide (SDEA) was associated with changes in astrocyte-associated responses after mTBI and in primary astroglial cultures. Methods: Male C57BL/6 mice received SDEA (10 mg/kg, subcutaneously) for 2 days before mTBI, immediately after injury, and for 7 days thereafter. At 11 weeks post-injury, cortical GFAP immunoreactivity and the expression of astrocyte-associated markers, PPAR isoforms, and neurotrophic factors were assessed by immunohistochemistry and RT-qPCR. Primary astroglial cultures were treated with 1 or 10 μM SDEA for 48 h. Results: SDEA attenuated mTBI-associated increases in GFAP-positive cells and cortical *Gfap* and *C3* expression while increasing *S100a10* and *Clcf1* expression. SDEA treatment was accompanied by increased cortical *Ppara* and *Pparg* mRNA expression and changes in the expression of neurotrophic-factor genes. In cultured astroglia, SDEA increased *S100a10*, *Clcf1*, *Ppara*, *Pparg*, *Bdnf*, *Ngf*, and *Gdnf* mRNA expression without significantly altering *Gfap* or *C3*. Conclusions: Peri-injury SDEA administration was associated with reduced chronic cortical GFAP reactivity and changes in selected astrocyte-associated transcripts after mTBI.

## 1. Introduction

Traumatic brain injury (TBI) affects an estimated 69 million people worldwide each year, including approximately 55.9 million cases classified as mild TBI (mTBI) [1]. Despite being classified as mild, mTBI can lead to persistent neurological and cognitive deficits [2,3]. This is due to the fact that, in addition to direct damage to brain tissue, mTBI triggers a complex cascade of pathological molecular and cellular processes that, over the long term, cause significant damage to the central nervous system (CNS). In this pathophysiological cascade, neuroinflammation is the primary contributor to the development of secondary brain damage and often determines the clinical outcome.

In turn, astrocytes, due to their multifunctional role in the CNS, are key participants in both physiological and pathological processes, including neuroinflammation [4,5,6,7]. Disruption of homeostasis in the CNS following traumatic injury causes the death of both neuronal and non-neuronal cells, leading to the release of DAMPs and the synthesis of a number of proinflammatory mediators, including IL1β, IL6, and TNFα. These factors, taken together, lead to molecular, functional, and morphological changes in astrocytes [8].

Reactive astrocytes can acquire diverse, context-dependent molecular and functional states that may include neurotoxic and neuroprotective features. The terms A1 and A2 have historically been used to describe inflammation-associated and reparative-associated transcriptional profiles, respectively; however, these categories do not represent fixed or mutually exclusive astrocyte phenotypes [9]. In the original A1 framework, microglia-derived IL-1α, TNF-α, and C1q induce a reactive astrocyte program characterized by increased expression of C3, CXCL10, and other inflammatory mediators. Such responses may sustain neuroinflammation, increase blood–brain barrier permeability through mediators including VEGF-A, VCAM-1, and matrix metalloproteinases, and contribute to secondary neuronal injury [7,8]. Other reactive astrocyte states are associated with mediators involved in inflammation resolution and tissue repair, including TGF-β, IGF-1, and IL-10 [7]. Reactive astrocytes may also express neurotrophic factors, including BDNF, NGF, and GDNF, which participate in neuronal survival, synaptic plasticity, and glia–neuron signaling [10,11,12]. These molecules are not exclusive markers of a particular astrocyte state, and changes in their expression alone cannot demonstrate an A1-to-A2 transition. Thus, modulation of astrocyte reactivity may represent a relevant experimental approach after TBI, but its effects should be described in terms of specific molecular and functional responses rather than a binary phenotype switch.

Compounds that occur endogenously in the central nervous system and participate in homeostatic and inflammatory regulation are of interest as experimental modulators of post-traumatic responses. Omega-3 polyunsaturated fatty acids (n-3 PUFAs) have a wide range of physiological effects and can influence various biological processes, including the nervous and immune systems [13]. Dietary n-3 PUFAs may influence the brain fatty acid (FA) profile and, as a consequence, the production of bioactive FA metabolites such as N-acylethanolamines (NAEs) [14], which are derivatives of FA linked via an amide bond to an ethanolamine group [15]. The most studied representative of this class of compounds is N-arachidonoylethanolamine (AEA), which is an endogenous mediator of the endocannabinoid system. Other NAEs, including N-palmitoylethanolamine (PEA) and N-oleoylethanolamine (OEA), are also considered bioactive lipid mediators involved in antinociceptive processes and the regulation of food intake. Other well-known NAEs, N-docosahexaenoylethanolamine (DHEA) and N-eicosapentaenoylethanolamine (EPEA), have anti-inflammatory and neuroprotective properties. However, among the representatives of this class, NAEs remain poorly studied, while neuroprotective effects have been repeatedly described for the fatty acid precursors of these compounds [16]. Stearidonic acid ethanolamide (SDEA) is an endogenous metabolite of the omega-3 PUFA stearidonic acid (SDA, C18:4 n-3). Our previous studies showed that, like other representatives of this class, SDEA exhibited anti-inflammatory and antioxidant properties, as well as improved the density and morphology of dendritic spines in the CA1 region of the hippocampus and partially restored proliferative activity in the dentate gyrus. Therefore, SDEA is a promising candidate for modulating neuroinflammation [17,18].

The present study examined whether peri-injury SDEA administration modifies chronic cortical GFAP immunoreactivity and selected reactive astrocyte-associated, PPAR, and neurotrophic-factor transcripts after mTBI.

## 2. Materials and Methods

### 2.1. Obtaining Stearidonic Acid Ethanolamide (SDEA)

N-stearidonylethanolamine used in this study was obtained from the fat of *Sardinops melanostictus*. The detailed procedure for the synthesis and purification of SDEA is presented in our previous publications [17,18]. Briefly, in the first step, ethyl esters were obtained from PUFAs, which were used to synthesize PUFA ethanolamides. Separation of the PUFA ethanolamide mixture was performed using preparative HPLC on a Shimadzu LC-8A system equipped with an SPD-20A UV/VIS detector (wavelength 205 nm) (Shimadzu Corporation, Kyoto, Japan). A Supelco Discovery HS C-18 reversed-phase column (Supelco, Bellefonte, PA, USA) (10 μm, 250 mm × 50 mm) was used for separation in isocratic mode using an ethanol-water mixture (70:30) at a flow rate of 50 mL/min. The target fractions corresponding to SDEA were collected, concentrated under vacuum, and analyzed by GC and GC-MS. The purity of the obtained compound was 94.8%.

### 2.2. Animals

The animal study protocol was approved by the Animal Ethics Committee of the A.V. Zhirmunsky National Scientific Center of Marine Biology, Far Eastern Branch of the Russian Academy of Sciences (protocol No. 2/2025, approved on 10 February 2025). All procedures were conducted in accordance with Directive 2010/63/EU and applicable national regulations. The study is reported in accordance with the ARRIVE 2.0 guidelines.

Male C57BL/6J mice were bred and maintained in the animal facility of the A.V. Zhirmunsky National Scientific Center of Marine Biology (Vladivostok, Russia). At the beginning of the experiment, the mice were approximately 3 months old and weighed 28–30 g. Because the animals were bred and maintained in the same facility, a separate post-transport acclimatization period was not required. Mice were housed four per cage under controlled conditions (22 ± 1 °C, 55 ± 15% relative humidity, 12 h light/dark cycle; lights on at 07:00), with food and water available ad libitum. Cages contained wood-shaving bedding and paper nesting material as environmental enrichment.

Animal health and welfare were assessed at least once daily throughout the experiment. On the day of the injury or sham procedure, mice were monitored continuously until recovery from anesthesia and were reassessed at approximately 2 and 24 h after the procedure. Prespecified humane endpoints included loss of more than 20% of body weight, persistent inability to ambulate or access food and water, prolonged recumbency, severe respiratory distress, recurrent or prolonged seizures, or signs of pain or distress that could not be relieved. No animal reached any of these endpoints.

Only male mice were included because the parent study was designed as an exploratory mechanistic experiment using the same sex as the authors’ previous mTBI studies. This maintained comparability with the existing experimental series and limited the number of animals required. Consequently, the study cannot address possible sex-dependent differences, which are acknowledged as a limitation.

### 2.3. mTBI Induction and Peri-Injury SDEA Administration

**Figure 1:**
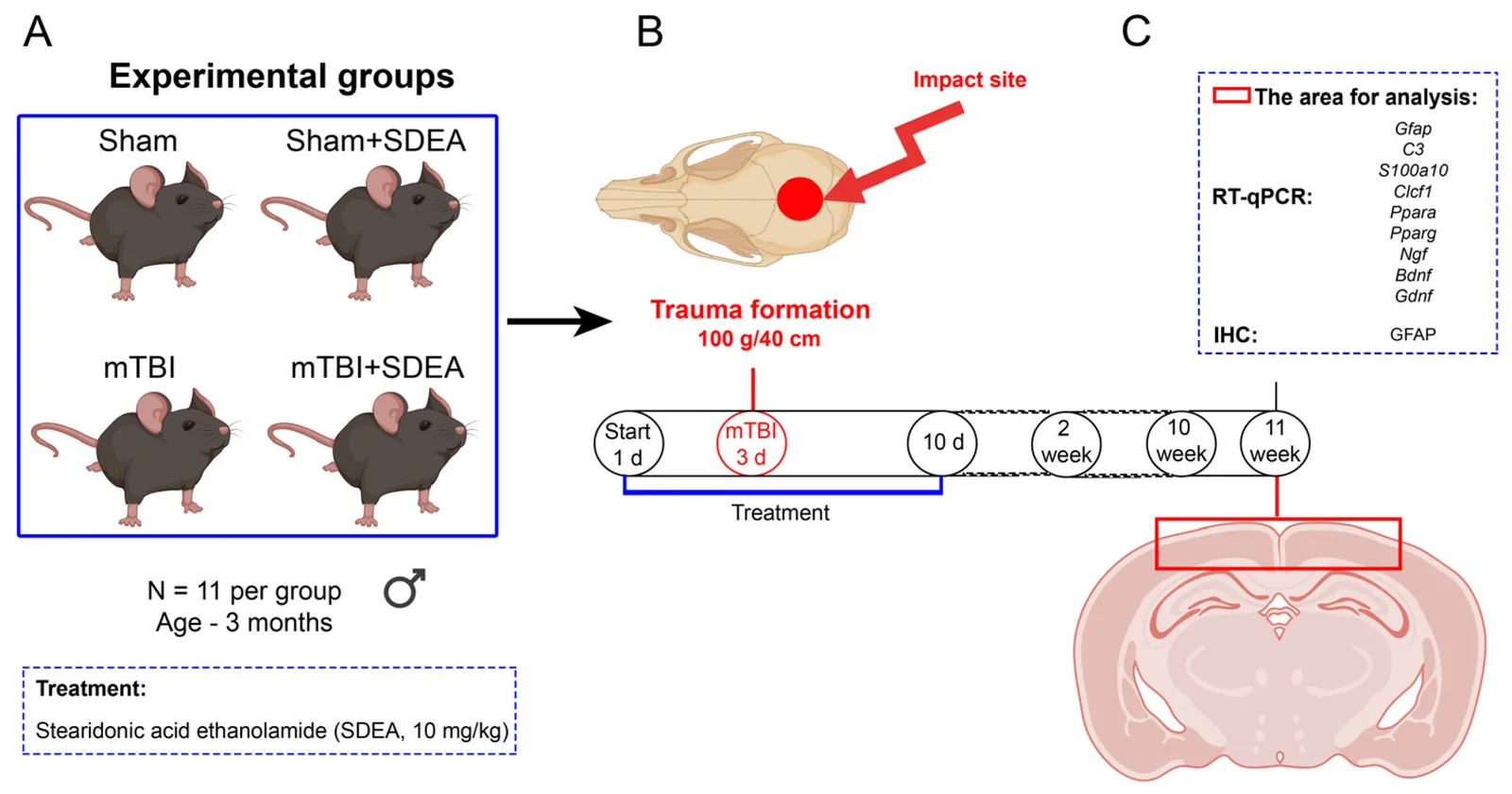
Experimental design. (**A**) Allocation of 44 mice to four experimental groups (*n* = 11 animals per group) and schedule of SDEA administration. At 11 weeks after injury, seven animals per group were used for immunohistochemistry and four animals per group were used for cortical RT-qPCR. (**B**) Procedure used to induce mTBI. (**C**) Cortical tissue sampling for immunohistochemical and molecular analyses. This figure presents the experimental design and does not contain quantitative comparisons.

The parent in vivo cohort comprised 68 mice assigned to four experimental groups, as reported previously [18]. Before outcome assessment, 44 mice from this cohort were prospectively allocated to the cortical analyses reported here. The animals were divided into four groups of 11 mice: “Sham” (sham procedure plus vehicle), “Sham+SDEA” (sham procedure plus SDEA), “mTBI” (mTBI plus vehicle), and “mTBI+SDEA” (mTBI plus SDEA). Within each group, seven mice were allocated to immunohistochemistry and four to cortical RT-qPCR (Figure 1A).

No formal a priori power calculation was performed, and no single outcome was designated as the primary outcome for determining sample size. Histological and transcriptional measurements were planned as complementary exploratory outcomes. Group sizes were based on the authors’ previous studies [17,18], the availability of tissue for complementary analyses, and the principle of reducing animal use while retaining biological replication. The SDEA dose of 10 mg/kg was selected on the basis of the same previous studies [17,18].

Group allocation was performed by simple randomization using a computer-generated random-number sequence. The allocation list was retained by an investigator who prepared the coded formulations but was not involved in outcome assessment. Animals, formulations, tissue samples, histological sections, images, and RT-qPCR files were identified using numerical codes. Investigators performing immunohistochemical quantification, RT-qPCR measurements, and statistical analyses remained unaware of group identity until the analyses had been completed. Because the injury and sham procedures were visually distinguishable, the investigator performing the impact could not be blinded to injury status.

Mice were anesthetized with 4.5% isoflurane (Karizoo, Barcelona, Spain) in 100% oxygen delivered using a VetFlo system (Kent Scientific, Torrington, CT, USA). After 1–3 min, when a stable level of anesthesia and loss of the withdrawal reflex had been confirmed, the scalp was shaved and a 1.25 mm thick protective metal plate was positioned over the parietal region. The center of the plate was aligned with the sagittal midline approximately 2.0 mm posterior to bregma (anteroposterior −2.0 mm; mediolateral 0.0 mm), corresponding approximately to the midpoint between bregma and lambda. The plate was temporarily secured to the shaved scalp using a small amount of tissue-compatible cyanoacrylate adhesive and was aligned beneath the guide tube before impact.

Each mouse was placed on a flexible polyurethane support mounted on the lower platform of an IMP-1020 impactor (Shanghai TOW Intelligent Technology Co., Ltd., Shanghai, China). A 100 g weight was raised to a height of 40 cm and released through the guide tube by an electromagnet. Under ideal free-fall conditions and neglecting friction and rebound, the estimated velocity of the weight immediately before impact was 2.80 m/s, and its gravitational potential energy was 0.392 J. These values describe the theoretical mechanics of the falling weight and should not be interpreted as the amount of energy transferred to the skull or brain.

The injury was classified as mild because it was produced by a single closed-head impact without craniotomy or macroscopically evident structural damage. Immediately after the procedure, animals were examined for skull fracture or deformation, convulsions, impaired spontaneous movement, abnormal posture, and other acute adverse events. No skull fracture, external cranial deformation, seizure activity, or mortality was observed. Acute neuroimaging, intracranial physiological monitoring, neurological severity scoring, and loss-of-righting-reflex measurements were not performed. Sham-operated mice underwent the same anesthesia, scalp preparation, metal-plate placement, positioning, and recovery procedures, but the weight was not released (Figure 1B).

After the injury or sham procedure, mice were transferred to a heated recovery surface and monitored continuously until spontaneous movement, normal posture, and thermoregulation had returned. They were reassessed approximately 2 and 24 h after the procedure and subsequently at least once daily. Neurological status was assessed at 24 h, 72 h, and 7 days after injury by evaluating spontaneous movement, posture, gait, coordination, and the presence of seizures or other overt neurological abnormalities.

SDEA and vehicle formulations were prepared immediately before each injection. SDEA was dispersed in water by vortexing at 3000 rpm for 5 min using a Multi-Vortex V-32 (BioSan, Riga, Latvia), after which ethanol was added to a final concentration of 1.5% (*v*/*v*). SDEA was administered subcutaneously at 10 mg/kg in a fixed volume of 100 μL per mouse, with the formulation concentration adjusted according to body weight. Vehicle-treated mice received 100 μL of water containing the same final ethanol concentration. Administration began 2 days before injury, continued immediately after the injury or sham procedure, and was repeated once daily for 7 days thereafter.

At 11 weeks after injury, seven mice per group underwent transcardial perfusion for immunohistochemistry, whereas four mice per group were euthanized without fixative perfusion for cortical RT-qPCR. The cerebral cortex was rapidly dissected, snap-frozen, and stored at −80 °C. No formal a priori inclusion or exclusion criteria were established. All 44 mice completed the protocol and were included in the prespecified datasets to which they had been allocated; there were no deaths, exclusions, or losses during follow-up. Measurements obtained from multiple sections of the same animal and technical replicates from the same biological sample were averaged before statistical analysis and were not treated as independent observations.

### 2.4. Immunohistochemical Studies and GFAP-Positive Cells’ Quantification

At 11 weeks post-surgery, animals were euthanized under deep anesthesia maintained with 4.5% isoflurane (Karizoo Laboratories, Barcelona, Spain) in 100% oxygen (VetFlo, Kent Scientific, Torrington, CT, USA) (Figure 1C). Transcardial perfusion was performed with ice-cold 0.1 M phosphate-buffered saline (PBS, pH 7.2), followed by fixation with chilled 4% paraformaldehyde in PBS (0.1 M, pH 7.2). After craniotomy, the brains were removed and post-fixed in fresh 4% paraformaldehyde for 24 h. The tissue was then washed in PBS, processed through standard histological protocols, and embedded in paraffin. A Leica RM 2245 rotary microtome (Leica, Wetzlar, Germany) was used to slice coronal sections (7 μm).

For immunohistochemical analysis, sections were first deparaffinized and then treated with 3% hydrogen peroxide. The sections were then washed in 0.1 M phosphate buffer (pH 7.2) and placed in a blocking solution containing 2% BSA (Sigma-Aldrich, Taufkirchen, Germany) and 0.01% Triton X-100 (Sigma-Aldrich, Taufkirchen, Germany) at room temperature for 1 h. The sections were then incubated with primary antibodies for 24 h at 4 °C: GFAP (1:1000; ab7260; Abcam, Cambridge, UK). Negative controls were processed similarly, but without primary antibodies. Following a 15 min treatment with the appropriate biotinylated anti-rabbit secondary antibody (ab64256, Abcam, Cambridge, UK), sections were incubated for 10 min with streptavidin-HRP (ab64269, Abcam, Cambridge, UK). DAB chromogen (ab64238, Abcam, Cambridge, UK) was used to visualize the sections. Sections were then cleared in xylene, dehydrated in a series of alcohols, and mounted using Dako mounting medium (CS705, Dako, Santa Clara, CA, USA).

The right dorsal parietal cortex beneath the impact site was analyzed. Coronal sections corresponding approximately to −1.70 to −1.94 mm relative to bregma were identified using a mouse brain atlas. Every sixth 7 μm section was selected, giving an interval of 42 μm between the analyzed sections. Six to seven sections were examined per mouse, and the same rostrocaudal range was used for all animals.

Bright-field images were acquired using a Zeiss Axio Imager microscope equipped with an AxioCam 503 color camera and ZEN Blue 3.4 software (Carl Zeiss, Oberkochen, Germany). Images used for quantification were acquired with a 20× objective at a resolution of 1936 × 1460 pixels. Higher-magnification images were obtained with a 40× objective. Exposure time, illumination, white balance and camera gain were fixed after initial adjustment and kept unchanged for all experimental groups. Images were converted to 8-bit format before analysis.

One rectangular region of interest measuring approximately 400 μm × 400 μm was placed in the dorsal cortex of each section. The ROI extended inward from the pial surface and was positioned using the same anatomical landmarks in all animals. Its position was not adjusted according to the intensity or extent of GFAP staining.

GFAP-positive cells were counted manually in ImageJ 1.53t by an investigator blinded to group allocation. A cell was counted when a clearly identifiable DAB-positive soma with adjoining GFAP-positive processes was visible. Isolated stained processes without a visible cell body were not counted. Cells were included when the center of the soma was located within the ROI.

The areal density of GFAP-positive cells was calculated as (N × 10^6^)/A, where N is the number of counted cells and A is the analyzed area in μm^2^. Measurements from six to seven sections were averaged to obtain one value for each mouse. Seven mice were analyzed per group, and the mouse was treated as the experimental unit. Individual-animal cortical GFAP immunohistochemistry data can be found in Supplementary Data S1.

### 2.5. Cell Culture

Primary astroglia-enriched cultures were prepared from the cerebral cortices of one-day-old (postnatal day 1, P1) C57BL/6 mouse pups. The neonatal pups were obtained separately from the adult mice used in the in vivo experiments. For each culture isolation, the cerebral cortices of four pups from a single litter were pooled. Tissue from different litters was not pooled. Three independent isolations were performed using pups from three separate litters. The sex of the pups was not determined at P1. Consequently, the cultures were considered to be derived from animals of undetermined and potentially mixed sex.

After decapitation, the cerebral cortices were dissected under sterile conditions, and the meninges were carefully removed. The pooled cortical tissue was minced, incubated in 0.25% trypsin (Gibco, Thermo Fisher Scientific, Waltham, MA, USA) in HBSS (HBSS; Gibco, Thermo Fisher Scientific, Waltham, MA, USA) for 30 min at 37 °C, and mechanically dissociated to obtain a single-cell suspension. Cells from each isolation were seeded into a poly-D-lysine-coated (Gibco, Thermo Fisher Scientific, Waltham, MA, USA) T75 flask and maintained in DMEM/F12 (Thermo Fisher Scientific, Waltham, MA, USA) supplemented with 10% fetal bovine serum (FBS; Gibco, Thermo Fisher Scientific, Waltham, MA, USA), 1% (*v*/*v*) MEM non-essential amino acids (100×; Gibco, Thermo Fisher Scientific), and 1% (*v*/*v*) penicillin–streptomycin (100×; Thermo Fisher Scientific), corresponding to final concentrations of 100 U/mL penicillin and 100 μg/mL streptomycin. Cultures were maintained at 37 °C in a humidified atmosphere containing 5% CO_2_. The medium was changed 48 h after plating and every 3 days thereafter.

At 7–8 days in vitro, the mixed glial cultures were shaken at 180 rpm for 30 min to remove loosely adherent microglia and then at 240 rpm for 6 h to reduce contamination by oligodendrocyte precursor cells. The remaining adherent cells were washed, detached with 0.05% trypsin-EDTA (Gibco, Thermo Fisher Scientific, Waltham, MA, USA), replated, and expanded for a further 12–14 days. Cells were then detached again and seeded into six-well plates at 1 × 10^5^ cells/cm^2^. Using the initial mixed culture as passage 0, the cells used for the experiments corresponded to passage 2 (P2) and were approximately 21–23 days in vitro at the beginning of treatment.

Immediately before treatment, SDEA was dispersed in complete culture medium and vortexed at 3000 rpm for 5 min using a Multi-Vortex V-32 (BioSan, Riga, Latvia). Ethanol was added stepwise to stabilize the dispersion, which was subsequently diluted with complete medium to final SDEA concentrations of 1 or 10 μM. All treatment and vehicle media contained 0.1% (*v*/*v*) ethanol and were prepared immediately before use. Vehicle-control cultures received complete medium containing 0.1% ethanol without SDEA. After 48 h, the cells were detached with 0.05% trypsin–EDTA, collected by centrifugation, frozen in liquid nitrogen, and stored until RNA isolation.

Each independent isolation was divided among the vehicle-control, 1 μM SDEA, and 10 μM SDEA conditions. Three independently prepared primary astroglial cultures obtained from separate cell isolations were used as biological replicates. Technical replicates within each independent culture were averaged before statistical analysis.

The SDEA concentrations were selected on the basis of our previous study, in which 10 μM SDEA did not reduce the viability of SIM-A9 microglial cells after 24 h of exposure, as assessed using the MTS assay [19]. Cell viability was not reassessed in the present primary astroglia-enriched cultures under the 48 h exposure protocol.

### 2.6. Reverse Transcription—Quantitative Polymerase Chain Reaction (RT-qPCR)

Total RNA was isolated from cerebral cortex (*n* = 4 mice per group; Figure 1C) and from astroglia-enriched primary cultures representing three independent isolations from three separate litters. Within each isolation, technical replicate wells were averaged before RT-qPCR analysis. Cortical tissue was homogenized using metal beads in a Tissue Lyser LT (Qiagen, Hilden, Germany). RNA was isolated using the ExtractRNA kit (Eurogen, Moscow, Russia) for cortex and the RNA Solo kit (BC034S, Evrogen, Moscow, Russia) with Lysis Buffer A containing β-mercaptoethanol for astroglia. RNA samples were treated with DNase I to remove genomic DNA (C12133, Magen, Guangzhou, China). Purification was performed with CleanRNA Standard (Eurogen, Moscow, Russia) for both sample types, and RNA quality was assessed spectrophotometrically (Thermo Fisher Scientific, Waltham, MA, USA). A260/A280 ranged from 2.0 to 2.1 and A260/A230 from 2.0 to 2.18, indicating high purity. First-strand cDNA was synthesized with the RNAscribe RT kit (Biolabmix, Novosibirsk, Russia) according to the manufacturer’s instructions.

RT-qPCR was performed using the SYBR Green I RT-PCR kit (Evrogen, Moscow, Russia) on a CFX96 thermocycler (Bio-Rad, Hercules, CA, USA) with the following thermal cycling conditions: 95 °C for 10 min (one cycle), then 95 °C for 15 s, 67 °C for 40 s, and 72 °C for 10 s (40 cycles), followed by melt-curve analysis. Amplicon sizes, primer sequences, and melting-curve data are provided in Supplementary Data S4. Melting curve analysis confirmed a single peak for each primer pair (Supplementary Data S4). The reaction was performed in a total volume of 20 μL with 1 μL of cDNA, and the primer concentration was 0.5 μM. Primers for the *Gfap* (glial fibrillary acidic protein), *C3* (complement c3), *S100a10* (s100 calcium-binding protein a10), *Clcf1* (cardiotrophin-like cytokine factor 1), *Ppara*, *Pparg* (peroxisome proliferator-activated receptor), *Bdnf* (brain-derived neurotrophic factor), *Ngf* (nerve growth factor), *Gdnf* (glial cell line-derived neurotrophic factor) genes were designed using Primer Premier 5 (Premier Biosoft International, Palo Alto, CA, USA) and synthesized by Evrogen (Evrogen, Moscow, Russia). Reference genes were *Gapdh* (glyceraldehyde-3-phosphate dehydrogenase) and *Actb* (beta-actin) (Table 1). No amplification was observed in NTC or NRT controls (undetermined Cq values), confirming the absence of reagent contamination and genomic DNA. Relative expression was calculated using the 2^−ΔΔCq^ method and is presented as fold change. The arithmetic mean of ΔCq values for Actb and Gapdh was used for normalization. Raw Cq values and ΔCq values are provided in Supplementary Datas S2 and S3.

### 2.7. Relationship with Previously Published Datasets

The relationship of the present work to the authors’ previous SDEA publications is summarized in Table S1. Ref. [17] used an independent adult cohort and distinct samples, images, molecular data, and cell experiments. The present in vivo analyses used a prespecified subset of the broader cohort reported in ref. [18]. Cortical immunohistochemistry was performed on the same brains and sections used previously for hippocampal analyses, but all cortical regions of interest, images, measurements, and numerical data are new. For RT-qPCR, statistical analyses were performed on animal- or culture-level ΔCq values, whereas relative expression calculated using the 2^−ΔΔCq^ method is presented for visualization. The cortical RT-qPCR measurements have not been published previously. The neonatal primary cultures were independent of both adult in vivo studies.

### 2.8. Statistical Analysis

Data were analyzed using GraphPad Prism 8.4.3 (GraphPad Software, Inc., San Diego, CA, USA). The individual animal was considered the experimental unit for all in vivo analyses, whereas an independently prepared primary astroglial culture was considered the experimental unit for the in vitro analyses. Measurements obtained from multiple sections of the same animal and technical RT-qPCR replicates from the same biological sample were averaged before statistical analysis.

For RT-qPCR, statistical analyses were performed using animal- or culture-level ΔCq values. Relative expression calculated using the 2^−ΔΔCq^ method is presented in the figures. In vivo data were analyzed by two-way ANOVA with injury and SDEA treatment as fixed factors. The main effects of injury and treatment and the injury × treatment interaction were evaluated separately. Tukey’s multiple-comparisons test was used for pairwise comparisons. In vitro ΔCq data were analyzed by one-way ANOVA followed by Tukey’s multiple-comparisons test.

Normality of model residuals was assessed using the Shapiro–Wilk test, and homogeneity of variance was evaluated using the Brown–Forsythe test. No significant violations of these assumptions were detected.

Cortical GFAP immunoreactivity was considered the primary histological outcome. The RT-qPCR marker panels were selected before unblinding and statistical analysis as secondary mechanistic outcomes. Tukey-adjusted *p* values were used for comparisons between experimental groups within each outcome. Data are presented as mean ± SEM, and *p* < 0.05 was considered statistically significant.

## 3. Results

### 3.1. SDEA Modifies Cortical GFAP Immunoreactivity and Selected Reactive Astrocyte-Associated Transcripts After mTBI

**Figure 2:**
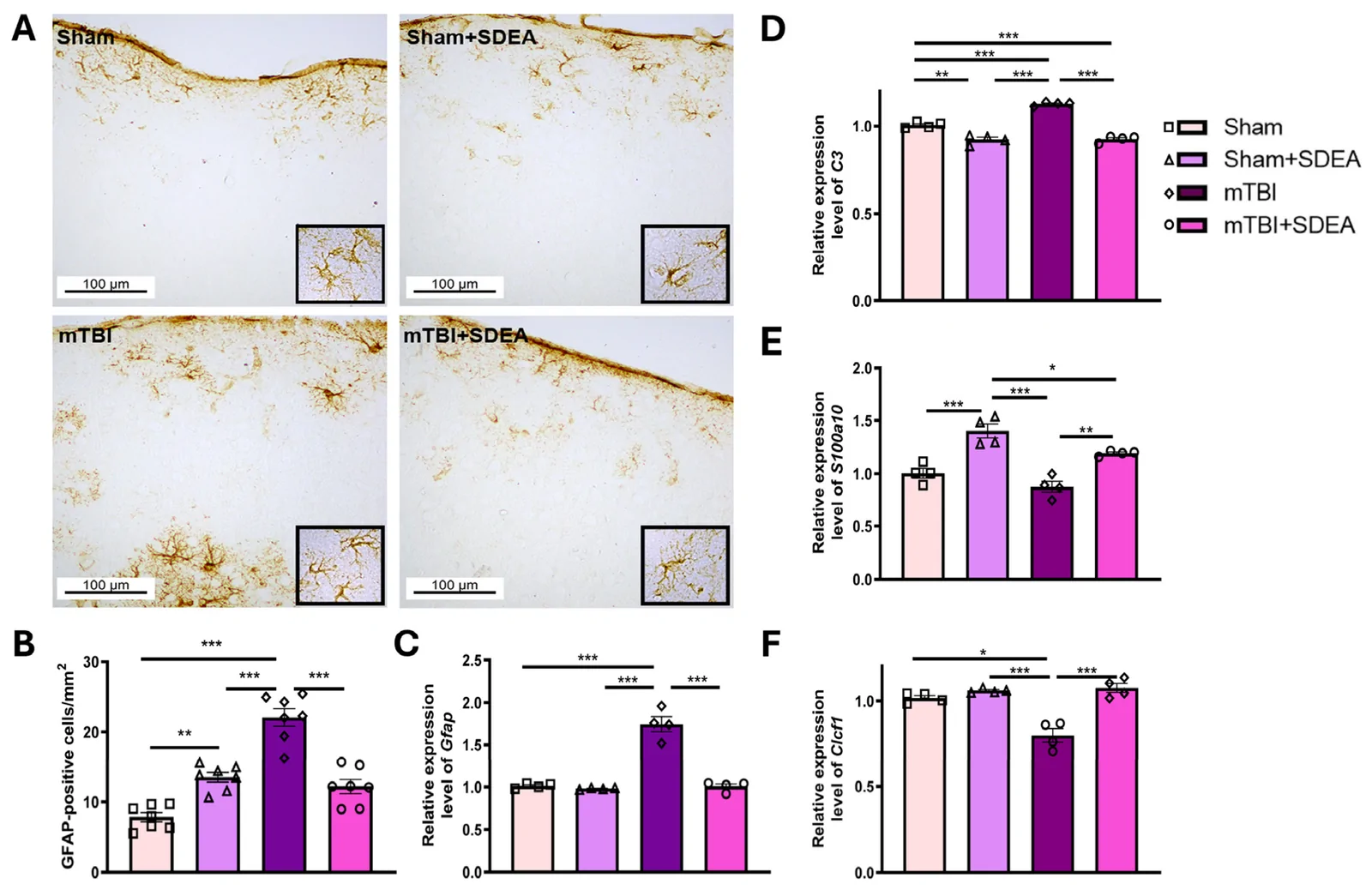
Cortical GFAP immunoreactivity and expression of selected reactive astrocyte-associated transcripts 11 weeks after mTBI. (**A**) Representative low-magnification images of GFAP immunohistochemistry in the cerebral cortex. Insets show corresponding higher-magnification views. Scale bars = 100 μm. (**B**) Two-dimensional areal density of GFAP-positive cells in the cerebral cortex, expressed as cells/mm^2^. Six to seven sections were analyzed per mouse and averaged to obtain one biological value. Seven mice were included in each group, and each point represents one mouse. (**C**) Relative mRNA expression of *Gfap*, (**D**) *C3*, (**E**) *S100a10*, and (**F**) *Clcf1* measured by RT-qPCR. Values are presented as mean ± SEM (relative expression normalized to housekeeping genes); *n* = 4 (animals per group). Two-way ANOVA, Tukey post hoc: * *p* < 0.05, ** *p* < 0.01, *** *p* < 0.001.

Neurological assessments performed at 24 h, 72 h, and 7 days after the injury or sham procedure revealed no overt deficits in any experimental group. To assess the long-term effect of SDEA on astroglial responses after mTBI, we first evaluated chronic astrogliosis by quantifying GFAP-positive cells in the cerebral cortex and then measured selected transcripts associated with reactive astrocyte responses. Glial fibrillary acidic protein (GFAP) is one of the key markers of astroglia. Traumatic brain injury and injury-induced neuroinflammation promote astroglial activation in the damaged brain regions, which is typically accompanied by increased GFAP expression. Therefore, elevated GFAP is considered a characteristic feature of both TBI and neurodegenerative disorders, including Parkinson’s disease and Alzheimer’s disease [20]. To determine whether SDEA treatment affects post-traumatic astroglial activation in the cerebral cortex during the chronic phase of mTBI, we quantified GFAP-positive cells in the cortical region (Figure 2A,B).

Immunohistochemical analysis demonstrated that mTBI increased the areal density of GFAP-positive cells in the cerebral cortex from 7.83 ± 0.65 cells/mm^2^ in the Sham group to 22.09 ± 1.25 cells/mm^2^ (Figure 2A,B). Two-way ANOVA showed significant effects of injury (F_1,24_ = 48.57, *p* < 0.0001), SDEA treatment (F_1,24_ = 5.19, *p* = 0.0319), and their interaction (F_1,24_ = 70.38, *p* < 0.0001). SDEA reduced GFAP-positive cell density in injured mice to 12.19 ± 1.00 cells/mm^2^ (*p* < 0.0001 vs. “mTBI”), although the value remained higher than in the “Sham” group (*p* = 0.0143). In sham-operated mice, SDEA increased GFAP-positive cell density to 13.50 ± 0.68 cells/mm^2^ (*p* = 0.0013 vs. “Sham”). No difference was found between the “Sham+SDEA” and “mTBI+SDEA” groups (*p* = 0.7485).

Whole-cortex RT-qPCR was used to examine *Gfap* and *C3*, which are associated with reactive and pro-inflammatory astroglial responses, and *S100a10* and *Clcf1*, which have been associated with reparative responses. These markers are nonexclusive and do not define discrete astrocyte phenotypes. Because RNA was isolated from whole cortical tissue, these measurements do not identify the cellular source of the transcripts.

The expression of *Gfap*, a marker of reactive astrogliosis, and *C3*, a transcript associated with pro-inflammatory astroglial responses, was analyzed to assess the pro-inflammatory astrocytic response (Figure 2C,D). For *Gfap*, two-way ANOVA revealed significant effects of injury (F_1,12_ = 137.74, *p* < 0.0001), SDEA treatment (F_1,12_ = 110.60, *p* < 0.0001), and their interaction (F_1,12_ = 95.50, *p* < 0.0001). Expression of *Gfap* increased markedly after mTBI (1.83 ± 0.06 vs. 1.00 ± 0.02 in “Sham”, *p* < 0.0001). SDEA treatment reduced *Gfap* expression in injured animals to 1.04 ± 0.04 (*p* < 0.0001 vs. “mTBI”). Expression in the “mTBI+SDEA” group did not differ from that in the “Sham” group (*p* = 0.8237), indicating restoration to the basal level. No significant difference was observed between the “Sham” and “Sham+SDEA” groups (*p* = 0.9511).

For *C3*, significant effects of injury (F_1,12_ = 37.68, *p* < 0.0001), treatment (F_1,12_ = 129.52, *p* < 0.0001), and their interaction (F_1,12_ = 24.85, *p* = 0.0003) were also detected. mTBI significantly increased *C3* expression (1.14 ± 0.01 vs. 1.00 ± 0.01 in “Sham”, *p* < 0.0001), whereas SDEA reduced its expression to 0.94 ± 0.02 (*p* < 0.0001 vs. “mTBI”). Interestingly, SDEA also significantly decreased *C3* expression in sham-operated animals (0.93 ± 0.01 vs. 1.00 ± 0.01 in “Sham”, *p* = 0.0034).

To evaluate reparative-associated transcriptional changes, the expression of *S100a10* and *Clcf1*, two transcripts previously associated with reparative astrocyte responses, was analyzed (Figure 2E,F). For *S100a10*, two-way ANOVA revealed significant effects of injury (F_1,12_ = 13.74, *p* = 0.0030) and SDEA treatment (F_1,12_ = 70.30, *p* < 0.0001), whereas their interaction was not significant (F_1,12_ = 1.74, *p* = 0.2118). mTBI alone did not significantly affect *S100a10* expression (0.93 ± 0.03 vs. 1.00 ± 0.05 in “Sham”, *p* = 0.3710). However, SDEA significantly increased its expression in injured animals to 1.18 ± 0.02 compared with both the “mTBI” (*p* = 0.0015) and “Sham” groups (*p* = 0.0277). A greater increase was observed in sham-operated mice receiving SDEA (1.41 ± 0.06), exceeding both the “Sham” (*p* < 0.0001) and “mTBI+SDEA” groups (*p* = 0.0180). Nevertheless, the absence of a significant injury × treatment interaction indicates that the magnitude of the response to SDEA was not shown to differ according to injury status.

Similarly, *Clcf1* expression was significantly affected by injury (F_1,12_ = 6.05, *p* = 0.0301), SDEA treatment (F_1,12_ = 28.07, *p* = 0.0002), and their interaction (F_1,12_ = 8.98, *p* = 0.0111). mTBI significantly reduced *Clcf1* expression (0.87 ± 0.04 vs. 1.01 ± 0.01 in “Sham”, *p* = 0.0106). SDEA treatment increased *Clcf1* expression in injured animals (“mTBI+SDEA”: 1.08 ± 0.02, *p* = 0.0004 vs. “mTBI”), restoring it to a level that did not differ significantly from the “Sham” group (*p* = 0.2385). No significant difference was detected between the “Sham” and “Sham+SDEA” groups (1.07 ± 0.01; *p* = 0.4005).

At 11 weeks after mTBI, cortical *Gfap* and *C3* expression was increased, whereas *Clcf1* expression was reduced. *S100a10* expression was not significantly altered by injury alone. Peri-injury SDEA administration reduced the injury-associated GFAP response, restored *Gfap* expression to the basal level, decreased *C3* expression, and increased *S100a10* and *Clcf1* expression in injured mice. SDEA also reduced *C3* and increased *S100a10* expression in sham-operated animals, indicating that its effects were not restricted to the injured cortex. These findings describe changes in selected reactive astrocyte-associated transcripts but do not establish conversion between discrete astrocyte phenotypes.

### 3.2. SDEA Alters Cortical Expression of PPAR and Neurotrophic-Factor Genes in the Mouse Cerebral Cortex After mTBI

**Figure 3:**
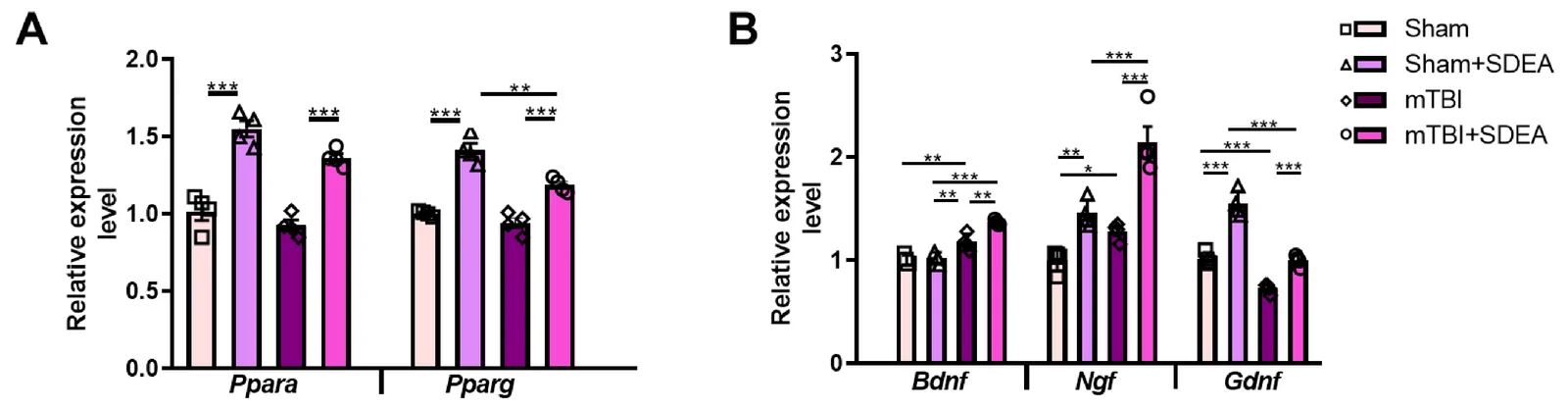
SDEA affects the expression of PPARs and neurotrophic factors in the mouse cerebral cortex after mTBI. (**A**) Relative mRNA expression of *Ppara* and *Pparg* in the cerebral cortex measured by RT-qPCR and normalized to the housekeeping genes *Actb* and *Gapdh*. (**B**) Relative mRNA expression of the neurotrophic factors *Bdnf*, *Ngf*, and *Gdnf* in the mouse cerebral cortex, measured by RT-qPCR and normalized to the reference genes *Actb* and *Gapdh*. Expression values are presented relative to the Sham group. Data are shown as mean ± SEM. Each point represents one animal (*n* = 4 mice per group). Statistical analysis was performed using two-way ANOVA with injury and SDEA treatment as factors, followed by Tukey’s multiple-comparisons test. * *p* < 0.05, ** *p* < 0.01, *** *p* < 0.001.

Peroxisome proliferator-activated receptors (PPARs) are nuclear receptors involved in the regulation of lipid metabolism, energy homeostasis, and inflammatory responses. Since SDEA belongs to the class of lipid mediators and the preceding results indicated its ability to modulate astroglial reactivity, we next examined whether SDEA treatment was associated with changes in the expression of *Ppara* and *Pparg* in the cerebral cortex after mTBI (Figure 3A).

Two-way ANOVA revealed that *Ppara* expression was significantly affected by injury (F_1,12_ = 6.30, *p* = 0.0274) and SDEA treatment (F_1,12_ = 91.37, *p* < 0.0001), whereas the injury × treatment interaction was not significant (F_1,12_ = 0.27, *p* = 0.6155). Pairwise comparisons showed that mTBI alone did not significantly alter *Ppara* expression compared with “Sham” animals (0.92 ± 0.04 vs. 1.01 ± 0.06, *p* = 0.5165). In contrast, SDEA markedly increased *Ppara* expression both in sham-operated animals (“Sham+SDEA”: 1.55 ± 0.05 vs. “Sham”, *p* < 0.0001) and in mice subjected to mTBI (“mTBI+SDEA”: 1.36 ± 0.03 vs. “mTBI”, *p* = 0.0002). Although *Ppara* expression was lower in the “mTBI+SDEA” group than in the “Sham+SDEA” group, the injury × treatment interaction was not significant. Therefore, the magnitude of the transcriptional response to SDEA cannot be considered statistically different between injury conditions.

For *Pparg*, two-way ANOVA showed significant effects of injury (F_1,12_ = 21.97, *p* = 0.0005) and SDEA treatment (F_1,12_ = 128.90, *p* < 0.0001), whereas the injury × treatment interaction was not significant (F_1,12_ = 4.27, *p* = 0.0611). Similar to *Ppara*, mTBI alone did not significantly change *Pparg* expression compared with “Sham” animals (0.94 ± 0.03 vs. 1.00 ± 0.01, *p* = 0.2972). However, SDEA significantly increased *Pparg* expression in both the “mTBI+SDEA” group (1.19 ± 0.02 vs. “mTBI”, *p* = 0.0001) and the “Sham+SDEA” group (1.41 ± 0.04 vs. “Sham”, *p* < 0.0001). The level of *Pparg* expression was significantly higher in “Sham+SDEA” animals than in “mTBI+SDEA” mice (*p* = 0.0022); however, the nonsignificant injury × treatment interaction does not establish that traumatic injury modified the transcriptional response to SDEA.

To determine whether the changes in PPAR expression were accompanied by transcriptional changes in neurotrophic factors, we quantified cortical *Bdnf*, *Ngf*, and *Gdnf* mRNA expression by RT-qPCR (Figure 3B).

For *Bdnf*, two-way ANOVA revealed significant effects of injury (F_1,12_ = 113.98, *p* < 0.0001), SDEA treatment (F_1,12_ = 10.31, *p* = 0.0075), and the injury × treatment interaction (F_1,12_ = 16.80, *p* = 0.0015). *Bdnf* expression was higher in the “mTBI” group than in the “Sham” group (1.18 ± 0.04 vs. 1.00 ± 0.03, *p* = 0.0027). SDEA further increased *Bdnf* expression in injured animals (“mTBI+SDEA”: 1.40 ± 0.02 vs. “mTBI”: 1.18 ± 0.04, *p* = 0.0012), whereas no difference was detected between the “Sham+SDEA” and “Sham” groups (0.98 ± 0.02 vs. 1.00 ± 0.03, *p* = 0.9211). The significant interaction indicates that the transcriptional response of *Bdnf* to SDEA depended on injury status.

For *Ngf*, significant main effects of injury (F_1,12_ = 37.68, *p* < 0.0001) and SDEA treatment (F_1,12_ = 69.90, *p* < 0.0001) were detected. The injury × treatment interaction was not statistically significant (F_1,12_ = 2.07, *p* = 0.1756). SDEA increased *Ngf* expression in both sham-operated animals (“Sham+SDEA”: 1.44 ± 0.07 vs. “Sham”: 1.01 ± 0.05, *p* = 0.0018) and injured mice (“mTBI+SDEA”: 2.14 ± 0.15 vs. “mTBI”: 1.28 ± 0.04, *p* < 0.0001). Following Tukey correction, *Ngf* expression was significantly higher in the “mTBI” group than in the “Sham” group (*p* = 0.0270). SDEA significantly increased cortical *Ngf* expression in both sham-operated and injured mice. Although the increase was numerically greater after mTBI, the injury × treatment interaction was not statistically significant (*p* = 0.1756).

For *Gdnf*, two-way ANOVA revealed significant effects of injury (F_1,12_ = 108.99, *p* < 0.0001) and SDEA treatment (F_1,12_ = 128.22, *p* < 0.0001), whereas their interaction was not significant (F_1,12_ = 0.51, *p* = 0.4867). mTBI reduced *Gdnf* expression compared with “Sham” animals (0.74 ± 0.03 vs. 1.00 ± 0.03, *p* < 0.0001). SDEA increased *Gdnf* expression in both sham-operated animals (“Sham+SDEA”: 1.46 ± 0.02 vs. “Sham”: 1.00 ± 0.03, *p* < 0.0001) and injured mice (“mTBI+SDEA”: 1.03 ± 0.04 vs. “mTBI”: 0.74 ± 0.03, *p* < 0.0001). *Gdnf* expression in the “mTBI+SDEA” group did not differ from that in the “Sham” group (*p* = 0.9221), indicating restoration to the basal level. However, it remained lower than in the “Sham+SDEA” group (*p* < 0.0001). Because the injury × treatment interaction was not significant, the data do not establish that injury attenuated the transcriptional response of *Gdnf* to SDEA.

Taken together, chronic mTBI was associated with increased cortical *Bdnf* and *Ngf* expression and reduced *Gdnf* expression. SDEA further increased *Bdnf* expression after mTBI, increased *Ngf* expression under both sham and injury conditions, and restored the mTBI-associated reduction in *Gdnf*. These transcriptional changes accompanied the increased expression of *Ppara* and *Pparg*.

### 3.3. SDEA Alters Selected Reactive Astrocyte-Associated Transcripts in Primary Astroglia-Enriched Cultures

To examine the response to SDEA under simplified culture conditions, astroglia-enriched primary cultures were exposed to 1 or 10 μM SDEA for 48 h, after which selected reactive astrocyte-associated transcripts were measured. Because the cellular composition of the cultures was not quantified, the results are attributed to the culture preparation as a whole rather than exclusively to astrocytes.

**Figure 4:**
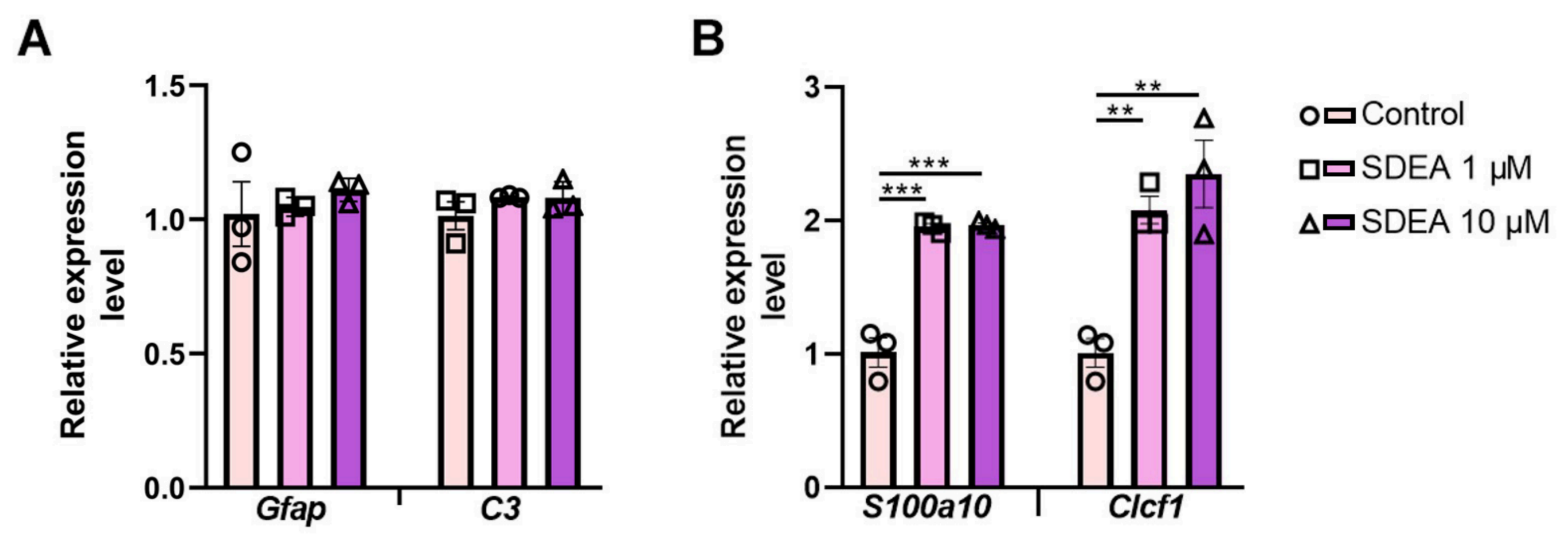
Effects of SDEA on selected reactive astrocyte-associated transcripts in primary astroglia-enriched cultures. (**A**) Relative expression of Gfap and C3. (**B**) Relative expression of S100a10 and Clcf1. Gene expression was measured by RT-qPCR and normalized to the housekeeping genes *Actb* and *Gapdh*. Data are presented as mean ± SEM. *n* = 3 independent culture isolations obtained from three separate litters. Each point represents one isolation. Technical replicate wells within an isolation were averaged before analysis. Statistical analysis was performed using one-way ANOVA followed by Tukey’s post hoc test. ** *p* < 0.01, *** *p* < 0.001.

SDEA did not significantly affect the expression of the *Gfap* and *C3*, which are associated with reactive and pro-inflammatory responses (Figure 4A). For *Gfap*, one-way ANOVA revealed no significant differences between groups (F_2,6_ = 0.560, *p* = 0.5984). The expression levels remained close to control values after treatment with both 1 μM SDEA (1.05 ± 0.02 vs. 1.01 ± 0.12 in the “control” group) and 10 μM SDEA (1.11 ± 0.03). Similarly, *C3* expression was not significantly altered by SDEA treatment (F_2,6_ = 1.221, *p* = 0.3589), with comparable levels observed in the “control” group (1.00 ± 0.06), SDEA 1 μM (1.08 ± 0.01), and SDEA 10 μM (1.08 ± 0.03) groups.

In contrast, SDEA markedly increased the expression of reparative-associated transcripts (Figure 4B). For *S100a10*, one-way ANOVA showed a significant effect of treatment (F_2,6_ = 33.709, *p* = 0.0005). Post hoc analysis demonstrated that both 1 μM and 10 μM SDEA significantly increased *S100a10* expression compared with the “control” group (1.96 ± 0.03 and 1.97 ± 0.02 vs. 1.01 ± 0.11, respectively; *p* < 0.001 for both comparisons). No significant difference was observed between the two SDEA concentrations.

A similar pattern was observed for *Clcf1*. One-way ANOVA revealed significant differences between groups (F_2,6_ = 22.483, *p* = 0.0016). SDEA significantly increased *Clcf1* expression at both 1 μM (2.08 ± 0.10, *p* = 0.0042 vs. “control”) and 10 μM (2.35 ± 0.26, *p* = 0.0020 vs. “control”), compared with the “control” group (1.01 ± 0.11). The difference between 1 μM and 10 μM SDEA was not statistically significant.

These results show that SDEA increased *S100a10* and *Clcf1* expression without detectable changes in *Gfap* or *C3*. They describe changes in selected transcripts and do not establish conversion to a fixed reparative astrocyte phenotype.

### 3.4. SDEA Increases PPAR and Neurotrophic-Factor Gene Expression in Primary Astroglia

Having shown that SDEA modulates astroglial activation, PPAR isoforms and neurotrophic-factor gene expression in the cerebral cortex after mTBI, we next investigated whether SDEA can alter selected transcripts under astroglia-enriched culture conditions, independently of the complex post-traumatic brain microenvironment.

**Figure 5:**
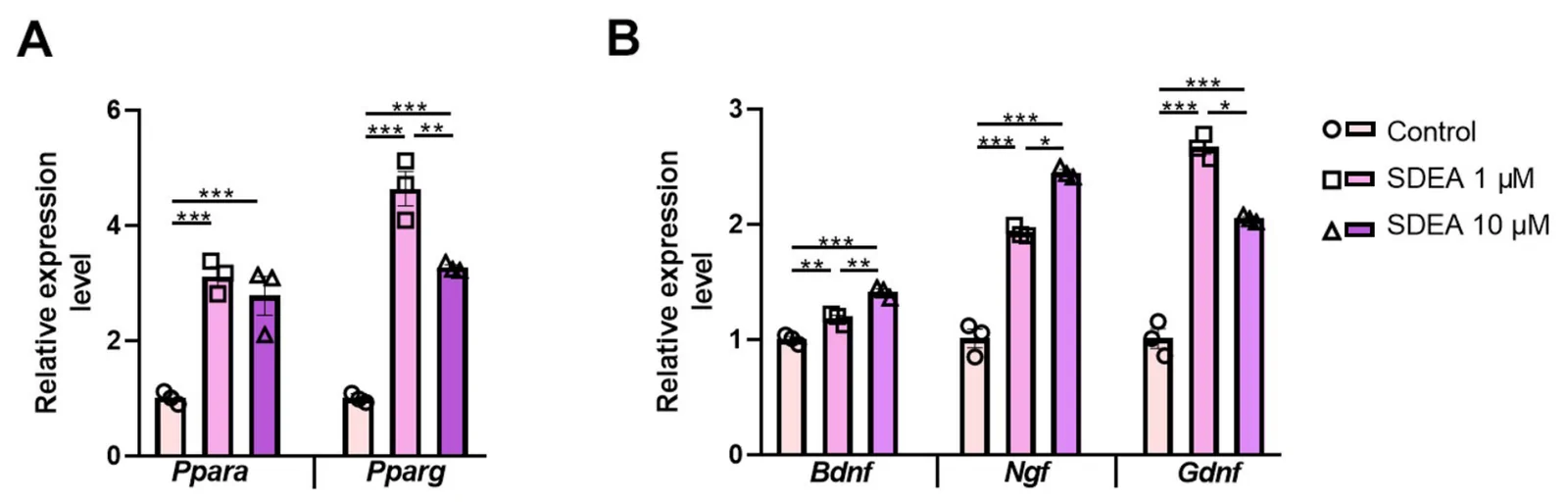
Effects of SDEA on the expression of genes encoding PPARs and neurotrophic factors in primary astroglia-enriched cultures. Primary astroglia cultures were treated with SDEA at concentrations of 1 and 10 μM. (**A**) Relative mRNA expression of *Ppara* and *Pparg*. (**B**) Relative mRNA expression of the neurotrophic factors *Bdnf*, *Ngf*, and *Gdnf*. Gene expression was measured by RT-qPCR and normalized to the housekeeping genes *Actb* and *Gapdh*. Data are presented as mean ± SEM. *n* = 3 independent culture isolations obtained from three separate litters. Each point represents one isolation. Technical replicate wells within each isolation were averaged before analysis. Statistical analysis was performed using one-way ANOVA followed by Tukey’s post hoc test. * *p* < 0.05, ** *p* < 0.01, *** *p* < 0.001.

According to RT-qPCR analysis, SDEA increased *Ppara* expression at both tested concentrations. Treatment with 1 μM SDEA increased *Ppara* expression approximately threefold (3.11 ± 0.16), whereas 10 μM SDEA increased it approximately 2.8-fold (2.76 ± 0.33), compared with the control group (1.00 ± 0.06; *p* < 0.001 for both comparisons). No significant difference was observed between the two concentrations. SDEA also markedly increased *Pparg* expression. The strongest effect was observed at 1 μM SDEA, where *Pparg* expression increased to 4.62 ± 0.30, whereas 10 μM SDEA increased expression to 3.26 ± 0.03, compared with “control” (1.00 ± 0.05; *p* < 0.0001 for both comparisons). Notably, *Pparg* expression was significantly lower at 10 μM than at 1 μM SDEA (*p* = 0.0050), suggesting a non-linear concentration-dependent response (Figure 5A).

We then evaluated whether SDEA affects the expression of neurotrophic factors in primary astroglia. One-way ANOVA revealed significant treatment effects for *Bdnf* (F_2,6_ = 61.72, *p* < 0.0001), *Ngf* (F_2,6_ = 77.05, *p* < 0.0001), and *Gdnf* (F_2,6_ = 88.13, *p* < 0.0001) (Figure 5B).

For *Bdnf*, both concentrations of SDEA significantly increased expression compared with “control” (1.18 ± 0.03 at 1 μM and 1.41 ± 0.03 at 10 μM vs. 1.00 ± 0.02 in “control”; *p* = 0.0040 and *p* < 0.001, respectively). Moreover, *Bdnf* expression was significantly higher after treatment with 10 μM SDEA than with 1 μM SDEA (*p* = 0.0030), indicating a concentration-dependent increase.

For *Ngf*, SDEA induced a robust increase at both concentrations. Expression increased from 1.01 ± 0.09 in “control” to 1.93 ± 0.03 after treatment with 1 μM SDEA and to 2.44 ± 0.03 after treatment with 10 μM SDEA (*p* < 0.001 vs. “control” for both comparisons). The 10 μM concentration induced significantly higher *Ngf* expression than 1 μM SDEA (*p* = 0.0448), indicating a concentration-dependent effect.

For *Gdnf*, SDEA also significantly increased expression at both concentrations. However, the maximal response was observed at 1 μM SDEA (2.67 ± 0.06), whereas 10 μM SDEA produced a smaller, although still significant, increase (2.05 ± 0.02), compared with “control” (1.01 ± 0.09; *p* < 0.001 for both comparisons). The level of *Gdnf* expression was significantly lower at 10 μM than at 1 μM SDEA (*p* = 0.0314), suggesting that the effect of SDEA on *Gdnf* follows a non-linear concentration-dependent pattern.

Overall, these in vitro results demonstrate that SDEA increases the expression of *Ppara*, *Pparg*, and neurotrophic factors in primary astroglia. Together with the induction of *S100a10* and *Clcf1*, these findings suggest that SDEA was associated with a reparative marker-expression profile.

## 4. Discussion

Astrocytes play a key role in the neuroinflammatory response that follows traumatic brain injury, functioning as active elements within a complex cellular network which also comprises microglia, neurons, endothelial cells and infiltrating immune cells [21]. Depending on the molecular context, reactive astrocytes may acquire neurotoxic or neuroprotective features and thereby either amplify tissue damage or contribute to the resolution of inflammation and tissue repair. In the present study, we show that chronic mTBI is associated with persistent astroglial reactivity in the cerebral cortex 11 weeks after injury. Importantly, SDEA treatment reduced cortical GFAP immunoreactivity after mTBI and altered the expression of selected transcripts associated with inflammatory and reparative responses. Because SDEA administration began before injury and continued during the early post-injury period, the findings should be interpreted as effects of peri-injury exposure rather than as treatment of established mTBI.

Reactive astrocytes may exhibit features historically described as A1- and A2-associated, although these categories are simplified descriptors within a broader spectrum of context-dependent astrocytic states [22]. A1-like responses can be induced by pro-inflammatory signals from microglia, including IL-1α, TNF-α, and C1q, and are associated with increased expression of complement components C3 and C4 and pro-inflammatory mediators such as IL-6 and CCL2 [23,24]. In contrast, A2-like responses have been associated with IL-4, IL-10, and TGF-β signaling, increased expression of markers such as S100A10 and CLCF1, and production of neurotrophic factors, including BDNF, GDNF, and NGF, which may contribute to neuroprotection, tissue repair, and resolution of inflammation [25]. However, these markers are nonexclusive and are not sufficient to define discrete astrocyte phenotypes or demonstrate a transition between them.

In line with previous studies demonstrating long-lasting astrocyte activation after TBI, mTBI increased cortical GFAP immunoreactivity and upregulated *Gfap* expression. This was accompanied by increased expression of *C3*, a transcript associated with complement activation and pro-inflammatory astroglial responses. At the same time, mTBI reduced *Clcf1* expression, whereas *S100a10* expression was not significantly altered by injury alone. Thus, rather than indicating a uniform change in all reparative-associated transcripts, our data suggest that chronic mTBI is accompanied by a predominantly reactive and pro-inflammatory-associated transcriptional pattern, with a selective reduction in *Clcf1* expression.

SDEA markedly modulated this response. In injured animals, SDEA reduced cortical GFAP immunoreactivity and normalized *Gfap* expression, indicating attenuation of post-traumatic reactive astrogliosis. SDEA also decreased *C3* expression and increased the reparative-associated transcripts *S100a10* and *Clcf1*. These findings suggest that SDEA does not simply suppress astroglial reactivity but modifies several components of the astroglial response. This interpretation is further supported by the effects observed in sham-operated animals: SDEA reduced *C3* expression and increased *S100a10*, while also increasing cortical GFAP immunoreactivity. Therefore, GFAP immunoreactivity alone should not be interpreted as a direct indicator of a neurotoxic response. The combined assessment of GFAP immunoreactivity and *Gfap*, *C3*, *S100a10*, and *Clcf1* expression provides a more complete description of the measured astroglial response. Together with the effects of SDEA on *Ppara*, *Pparg*, *Ngf*, and *Gdnf* expression in sham-operated animals, these findings indicate that SDEA has biological activity in the absence of injury. Thus, the similarity between some measurements in the “Sham+SDEA” and “mTBI+SDEA” groups should not be interpreted solely as normalization of post-traumatic changes but may reflect broader effects of SDEA under both experimental conditions. Accordingly, an effect was considered injury dependent only when supported by a significant injury and treatment interaction.

Because SDEA is a lipid mediator derived from omega-3 polyunsaturated fatty acids, we next examined whether its effects were associated with changes in PPAR expression. PPAR-α and PPAR-γ are nuclear receptors involved in lipid metabolism, energy homeostasis, and resolution of inflammation. Activation of PPAR-α and PPAR-γ has been reported to suppress pro-inflammatory gene expression and induce neurotrophic factors [26,27]. In our study, mTBI alone did not significantly alter cortical *Ppara* or *Pparg* mRNA expression compared with sham-operated animals. SDEA increased the expression of both genes in sham-operated and injured mice, with no clear evidence that the response depended on injury status.

These findings indicate an association between SDEA treatment and the expression of genes related to PPAR signaling. This association is biologically plausible, since traumatic brain injury is accompanied by membrane disruption and depletion of polyunsaturated fatty acids, including docosahexaenoic acid, which may enhance neuroinflammation and oxidative stress and potentially reduce endogenous stimulation of PPAR-related pathways [28,29,30]. However, increased *Ppara* and *Pparg* mRNA levels do not by themselves prove functional activation of PPAR-dependent pathways. Direct confirmation would require additional experiments assessing PPAR protein levels, receptor activity, downstream transcriptional targets, or the use of selective PPAR antagonists. Therefore, the present data show that SDEA treatment is associated with increased *Ppara* and *Pparg* mRNA expression, but do not establish that PPAR activation mediates its anti-inflammatory or pro-reparative effects.

RT-qPCR showed that mTBI and SDEA treatment affected the cortical expression of *Bdnf*, *Ngf*, and *Gdnf* differently. At the chronic time point examined, mTBI increased *Bdnf* and *Ngf* expression and reduced *Gdnf* expression. Thus, chronic injury did not produce a uniform neurotrophic response but differentially affected individual components of this system.

The increase in *Bdnf* expression after mTBI may reflect an adaptive response to persistent post-traumatic changes. SDEA produced a further increase in injured animals but had no significant effect in sham-operated mice. The significant injury and treatment interaction indicates that the effect of SDEA on *Bdnf* transcription depended on injury status. Both BDNF and NGF have been shown to modulate inflammatory responses in experimental systems [31,32,33,34], while changes in BDNF and GDNF have accompanied neuroplastic and neuroprotective adaptations in other animal models [35]. In addition, TNF-α can induce *Bdnf* transcription and BDNF production in primary astrocytes [36], suggesting that increased cortical *Bdnf* expression after mTBI may form part of an inflammation-associated compensatory response. However, the relationship between BDNF and neuroinflammation is bidirectional and depends on the cellular and pathological context [37]. Therefore, increased *Bdnf* expression should not be interpreted as evidence of enhanced neuroprotection.

*Ngf* expression was moderately increased after mTBI and rose further following SDEA administration. SDEA also enhanced *Ngf* expression in sham-operated mice, indicating that its effect was not restricted to the injured cortex. Overall, the findings point to a general stimulatory effect of SDEA on cortical *Ngf* expression rather than a response specific to the post-traumatic state. Its functional significance may be context dependent and should not be interpreted as direct evidence of neuroprotection.

In contrast to *Bdnf* and *Ngf*, chronic mTBI reduced cortical *Gdnf* expression. SDEA reversed this reduction and restored *Gdnf* expression to the level observed in sham-operated animals. Because SDEA also affected *Gdnf* expression in the absence of injury, this finding should not be interpreted exclusively as reversal of an mTBI-induced change. Moreover, the increase in *Gdnf* mRNA does not by itself demonstrate a corresponding increase in GDNF protein or neuroprotective activity.

The in vivo findings were broadly consistent with the results obtained in primary astroglia-enriched cultures, in which SDEA increased *Bdnf*, *Ngf*, and *Gdnf* expression. This agreement suggests that astroglia may contribute to the transcriptional response observed in the cerebral cortex. However, the in vivo measurements were performed in whole cortical tissue and cannot identify the cellular source of the detected transcripts. Similarly, the culture experiments show that SDEA induces these transcriptional changes in astroglia-enriched cultures but do not allow them to be attributed exclusively to astrocytes. Moreover, increased neurotrophic-factor mRNA expression does not by itself demonstrate a corresponding increase in protein abundance or neuroprotective activity.

The changes in neurotrophic-factor gene expression occurred together with increased cortical *Ppara* and *Pparg* mRNA expression. PPAR signaling has previously been linked to the regulation of neurotrophic genes, including *Bdnf* [27]. These observations indicate a possible association with PPAR-related signaling, although they do not establish that PPAR activation mediated the effects of SDEA. Confirmation of such a mechanism would require assessment of PPAR protein levels, receptor activity, downstream transcriptional targets, or experiments using selective PPAR antagonists.

The effects of SDEA on *Gfap* and *C3* differed between the cerebral cortex and the astroglia-enriched cultures. SDEA reduced both transcripts in vivo but did not alter their expression in cultures maintained without an injury-related or pro-inflammatory stimulus. This difference may reflect the influence of the post-traumatic tissue environment, including signaling between astrocytes, microglia, neurons, endothelial cells, and infiltrating immune cells. It is also possible that SDEA affected cortical *Gfap* and *C3* expression indirectly through other cell populations. Therefore, the in vivo changes cannot be attributed exclusively to a direct action of SDEA on astrocytes. At the same time, the increases in *S100a10*, *Clcf1*, PPAR isoforms, and neurotrophic-factor transcripts in culture show that the astroglia-enriched preparation was capable of responding directly to SDEA.

Taken together, our in vivo and in vitro data suggest that peri-injury SDEA attenuates the pro-inflammatory astroglial response after mTBI and enhances the expression of genes associated with reparative astrocyte functions and neurotrophic support. The effects of SDEA were accompanied by increased *Ppara* and *Pparg* mRNA expression. Although this association identifies PPAR-related signaling as a potential subject for further investigation, it does not establish PPAR activation or a causal role for these receptors.

Several limitations should be considered. First, the study was performed at a single chronic time point after mTBI. Therefore, the temporal course of the astroglial response to SDEA remains unknown. SDEA administration began two days before injury, and the experimental design consequently evaluates peri-injury modulation rather than treatment initiated after established mTBI. This regimen ensured exposure at the time of injury but does not reproduce the usual clinical situation, in which treatment can begin only after an unexpected mTBI. Further experiments using exclusively post-injury administration are required to determine the therapeutic potential and clinically relevant treatment window of SDEA.

In addition, only male mice were used. Because sex can influence neuroinflammatory and post-traumatic responses, the present findings cannot be generalized to females, and future studies should include animals of both sexes.

The molecular findings also require cautious interpretation. Only a limited panel of nonexclusive reactive astrocyte-associated transcripts was examined, and these markers cannot define a discrete astrocyte phenotype. Because RT-qPCR was performed using whole-cortex samples, the cellular sources of the measured transcripts could not be determined. Moreover, changes in transcript abundance do not necessarily correspond to changes in protein levels or biological activity. PPAR protein expression, receptor activity, and downstream signaling were not assessed, and selective PPAR antagonists were not used.

Although 10 μM SDEA was not cytotoxic to SIM-A9 microglial cells in our previous study, viability was not assessed directly in the primary astroglia-enriched cultures examined here.

Although SDEA was the predominant component of the preparation, the possible contribution of the remaining minor constituents cannot be excluded. Confirmation using SDEA of higher purity and a fully characterized impurity profile is therefore required.

Finally, the study did not include behavioral or electrophysiological assessment or protein-level outcomes. It therefore remains unknown whether the observed molecular and histological changes are accompanied by functional improvement, and the present findings should not be regarded as evidence of therapeutic efficacy.

## 5. Conclusions

In conclusion, the present study demonstrates that peri-injury SDEA modulates astroglial reactivity in the chronic phase after mTBI. SDEA administration was associated with reduced chronic cortical GFAP reactivity after mTBI and with changes in selected transcripts related to reactive astroglial, inflammatory, and reparative responses. Several SDEA-associated changes were also observed in sham-operated animals, indicating that its effects were not restricted to the post-traumatic state. The increase in *Ppara* and *Pparg* mRNA expression identifies PPAR-related signaling as a possible associated pathway, but does not demonstrate receptor activation or mediation of the observed effects.

## Figures and Tables

**Table 1:** PCR primers used in the study.

Name	Forward Seq	Reverse Seq
*Gapdh*	CATTGTGGAAGGGCTCA	TAGGGACGTAGGTGACC
*Actb*	GGCTGTATTCCCCTCCATCG	AGTCCTTCTGACCCATTCCCAC
*Gfap*	ATGTGTCCCCAGAGTTCTATTG	TTCACTACAAGAGCAGCCGTC
*C3*	GCCTCTCCTCTGACCTCTGG	AGTTCTTCGCACTGTTTCTGG
*S100a10*	CAAAATAATGAAGGACCTGGACC	ACAAGAAGCAGTGGGGCAGATT
*Clcf1*	AAACCTATGACCTCACCCGCTAC	GCCACACTTCCAAGTTGACCG
*Gdnf*	AGACTGAAAAGGTCACCAGATAAA	CATAGCCCAAACCCAAGTCAGT
*Ngf*	ACAGACATCAAGGGCAAGGAGG	ACAGGATTGGAGGCTCGGC
*Bdnf*	GGCTGACACTTTTGAGCACGTC	CTCCAAAGGCACTTGACTGCTG
*Ppara*	AAGTGCCTGTCTGTCGGGATGT	TCTTCAGGTAGGCTTCGTGGATT
*Pparg*	GCCCTTTACCACAGTTGATTTCTC	TCGCACTTTGGTATTCTTGGAGC

## Data Availability

The data supporting the findings of this study are contained within the article and its Supplementary Materials. Raw Cq values, ΔCq values used for statistical analysis, relative-expression data, and individual-animal immunohistochemistry data are provided in Supplementary Materials Data S1–S4. Further inquiries can be directed to the corresponding author.

## Supplementary Materials

The following supporting information can be downloaded at https://www.mdpi.com/article/10.3390/pathophysiology33040078/s1, Table S1: Relationship between the present dataset and the authors’ previously published studies; Supplementary Data S1: Individual-animal cortical GFAP immunohistochemistry data; Supplementary Data S2: Raw and processed RT-qPCR data from the mouse cerebral cortex; Supplementary Data S3: Raw and processed RT-qPCR data from primary astroglia-enriched cultures; Supplementary Data S4: RT-qPCR primer sequences, amplicon lengths, and melting curves; Supplementary S5: The ARRIVE guidelines 2.0: author checklist.

## Abbreviations

The following abbreviations are used in this manuscript:

mTBI	mild traumatic brain injury
CNS	central nervous system
PUFA	polyunsaturated fatty acid
NAE	N-acylethanolamide
SDEA	stearidonic acid ethanolamide
DHEA	N-docosahexaenoylethanolamine
EPEA	N-eicosapentaenoylethanolamine
SDA	stearidonic acid
GFAP	glial fibrillary acidic protein
PPAR	peroxisome proliferator-activated receptors
C3	Complement component 3
S100A10	S100 calcium-binding protein A10
CLCF1	cardiotrophin-like cytokine factor 1
BDNF	Brain-Derived Neurotrophic Factor
NGF	Nerve Growth Factor
GDNF	Glial Cell Line Derived Neurotrophic Factor
IL-1β	interleukin-1 beta
IL-6	interleukin-6
IL-4	interleukin-4
IL-10	interleukin-10
TNF-α	tumor necrosis factor alpha
NF-κB	Nuclear factor kappa-light-chain-enhancer of activated B cells
TGF-β	Transforming growth factor beta
RT-PCR	reverse transcription quantitative polymerase chain reaction
SEM	standard error of the mean
ANOVA	analysis of variance
