# Supplementary material for: Peri-Injury Stearidonic Acid Ethanolamide Modulates Astroglial Reactivity-Associated Markers in a Mouse Model of Mild Traumatic Brain Injury
Source: Pathophysiology. 2026 Oct 20;33(4):78. doi: 10.3390/pathophysiology33040078 (PMC13692413; doi:10.3390/pathophysiology33040078)
Supplement: Supplementary file 1 [file pathophysiology-33-00078-s001.zip › Supplementary S5 The ARRIVE guidelines 2.0 author checklist.pdf]

NOTE: Please save this file locally before filling in the table, DO NOT work on the file within your internet browser as changes will not be saved. Adobe Acrobat Reader (available free here) is recommended for completion.

The ARRIVE guidelines 2.0: author checklist

The ARRIVE Essential 10

These items are the basic minimum to include in a manuscript. Without this information, readers and reviewers cannot assess the reliability of the findings.

Item		Recommendation	Section/line number, or reason for not reporting
**Study design**	1	For each experiment, provide brief details of study design including:	
		a. The groups being compared, including control groups. If no control group has been used, the rationale should be stated.	Methods 2.3, p. 3, lines 131 – 137; Figure 1, p. 5, lines 206 – 211.
		b. The experimental unit (e.g. a single animal, litter, or cage of animals).	Methods 2.8, p. 8, lines 353 – 358; p. 7, lines 296 – 299.
**Sample size**	2	a. Specify the exact number of experimental units allocated to each group, and the total number in each experiment. Also indicate the total number of animals used.	Methods 2.3, p. 3, lines 131 – 137; p. 5, lines 194 – 202. Methods 2.5, p. 6, lines 263 – 269; p. 7, lines 296 – 299.
		b. Explain how the sample size was decided. Provide details of any *a priori* sample size calculation, if done.	p. 3, lines 138 – 140; p. 4, lines 141 – 144.
**Inclusion and exclusion criteria**	3	a. Describe any criteria used for including and excluding animals (or experimental units) during the experiment, and data points during the analysis. Specify if these criteria were established *a priori*. If no criteria were set, state this explicitly.	Methods 2.3, p. 5, lines 194 – 202
		b. For each experimental group, report any animals, experimental units or data points not included in the analysis and explain why. If there were no exclusions, state so.	Methods 2.3, p. 5, lines 194 – 202
		c. For each analysis, report the exact value of *n* in each experimental group.	Figure 2, p. 10, lines 405 – 410; Figure 3, p. 13, lines 548 – 551; Figure 4, p. 14, lines 587 – 591; Figure 5, p. 15, lines 641 – 645..
**Randomisation**	4	a. State whether randomisation was used to allocate experimental units to control and treatment groups. If done, provide the method used to generate the randomisation sequence.	Methods 2.3, p. 4, lines 145 – 147..
		b. Describe the strategy used to minimise potential confounders such as the order of treatments and measurements, or animal/cage location. If confounders were not controlled, state this explicitly.	Methods 2.3.
**Blinding**	5	Describe who was aware of the group allocation at the different stages of the experiment (during the allocation, the conduct of the experiment, the outcome assessment, and the data analysis).	Methods 2.3, p. 4, lines 145 – 153; Methods 2.4, p. 6, lines 252 – 253.
**Outcome measures**	6	a. Clearly define all outcome measures assessed (e.g. cell death, molecular markers, or behavioural changes).	Methods 2.3, p. 4, lines 145 – 147.
		b. For hypothesis-testing studies, specify the primary outcome measure, i.e. the outcome measure that was used to determine the sample size.	p. 9, lines 369 – 371; p. 3, lines 138 – 140.
**Statistical methods**	7	a. Provide details of the statistical methods used for each analysis, including software used.	Methods 2.8, p. 8, lines 353 – 365; p. 9, lines 369 – 373.
		b. Describe any methods used to assess whether the data met the assumptions of the statistical approach, and what was done if the assumptions were not met.	p. 8, lines 366 – 368.
**Experimental animals**	8	a. Provide species-appropriate details of the animals used, including species, strain and substrain, sex, age or developmental stage, and, if relevant, weight.	Methods 2.2, p. 3, lines 109 – 116, 124 – 128. Methods 2.5, p. 6, lines 263 – 269.
		b. Provide further relevant information on the provenance of animals, health/immune status, genetic modification status, genotype, and any previous procedures.	p. 3, lines 109 – 113; p. 3, lines 131 – 137; p. 8, lines 340 – 350.
**Experimental procedures**	9	For each experimental group, including controls, describe the procedures in enough detail to allow others to replicate them, including:	
		a. What was done, how it was done and what was used.	Methods 2.3 – 2.6: p. 4, lines 154 – 191; p. 5, lines 192 – 202, 214 – 229; p. 6, lines 230 – 280; p. 7, lines 281 – 331; p. 8, lines 332 – 336.
		b. When and how often.	p. 4, lines 180 – 185; p. 5, lines 192 – 197; p. 7, lines 281 – 303
		c. Where (including detail of any acclimatisation periods).	p. 3, lines 109 – 116.
		d. Why (provide rationale for procedures).	p. 2, lines 80 – 88; p. 4, lines 143 – 144; p. 7, lines 300 – 303.
**Results**	10	For each experiment conducted, including independent replications, report:	
		a. Summary/descriptive statistics for each experimental group, with a measure of variability where applicable (e.g. mean and SD, or median and range).	Results 3.1 – 3.4, pp. 9 – 15.: p. 10, lines 401 – 410; p. 13, lines 543 – 551; p. 14, lines 584 – 591; p. 15, lines 636 – 645.
		b. If applicable, the effect size with a confidence interval.	Effect sizes for the main effects and interaction were expressed as partial eta squared (ηp^2). For pairwise comparisons, effect sizes are presented as estimated mean differences with 95% confidence intervals.

The Recommended Set

These items complement the Essential 10 and add important context to the study. Reporting the items in both sets represents best practice.

Item		Recommendation	Section/line number, or reason for not reporting
**Abstract**	11	Provide an accurate summary of the research objectives, animal species, strain and sex, key methods, principal findings, and study conclusions.	Ap. 1, lines 12 – 27.
**Background**	12	a. Include sufficient scientific background to understand the rationale and context for the study, and explain the experimental approach.	p. 1, lines 32 – 39; p. 2, lines 40 – 85.
		b. Explain how the animal species and model used address the scientific objectives and, where appropriate, the relevance to human biology.	p. 1, lines 32 – 39; p. 2, lines 80 – 88; p. 4, lines 163 – 179.
**Objectives**	13	Clearly describe the research question, research objectives and, where appropriate, specific hypotheses being tested.	Introduction, p. 2, lines 86 – 88..
**Ethical statement**	14	Provide the name of the ethical review committee or equivalent that has approved the use of animals in this study, and any relevant licence or protocol numbers (if applicable). If ethical approval was not sought or granted, provide a justification.	Methods 2.2, p. 3, lines 104 – 108; Institutional Review Board Statement, p. 19, lines 842 – 845.
**Housing and husbandry**	15	Provide details of housing and husbandry conditions, including any environmental enrichment.	Methods 2.2, p. 3, lines 109 – 116.
**Animal care and monitoring**	16	a. Describe any interventions or steps taken in the experimental protocols to reduce pain, suffering and distress.	p. 4, lines 154 – 157; p. 4, lines 180 – 185; p. 5, lines 214 – 216.
		b. Report any expected or unexpected adverse events.	p. 4, lines 171 – 177; p. 5, lines 198 – 200.
		c. Describe the humane endpoints established for the study, the signs that were monitored and the frequency of monitoring. If the study did not have humane endpoints, state this.	p. 3, lines 117 – 123; p. 4, lines 180 – 185.
**Interpretation/ scientific implications**	17	a. Interpret the results, taking into account the study objectives and hypotheses, current theory and other relevant studies in the literature.	Discussion, p. 15, lines 648 – 666; p. 16, lines 667 – 716; p. 17, lines 717 – 766; p. 18, lines 767 – 791.
		b. Comment on the study limitations including potential sources of bias, limitations of the animal model, and imprecision associated with the results.	Study limitations, p. 18, lines 792 – 816; p. 19, lines 817 – 820.
**Generalisability/ translation**	18	Comment on whether, and how, the findings of this study are likely to generalise to other species or experimental conditions, including any relevance to human biology (where appropriate).	p. 18, lines 794 – 800. p. 18, lines 801 – 803. p. 19, lines 817 – 820.
**Protocol registration**	19	Provide a statement indicating whether a protocol (including the research question, key design features, and analysis plan) was prepared before the study, and if and where this protocol was registered.	Not reported
**Data access**	20	Provide a statement describing if and where study data are available.	ata Availability Statement, p. 19, lines 847 – 85[illegible]
**Declaration of interests**	21	a. Declare any potential conflicts of interest, including financial and non-financial. If none exist, this should be stated.	Conflicts of Interest, p. 19, lines 851 – 852.
		b. List all funding sources (including grant identifier) and the role of the funder(s) in the design, analysis and reporting of the study.	Funding, p. 19, line 841.
