# Supplementary material for: Peri-Injury Stearidonic Acid Ethanolamide Modulates Astroglial Reactivity-Associated Markers in a Mouse Model of Mild Traumatic Brain Injury
Source: Pathophysiology. 2026 Oct 20;33(4):78. doi: 10.3390/pathophysiology33040078 (PMC13692413; doi:10.3390/pathophysiology33040078)
Supplement: Supplementary file 1 [file pathophysiology-33-00078-s001.zip › Table S1.pdf]

Relationship to previously published datasets

The relationship of the present work to the authors' previous SDEA publications is summarized in Table 1. Ref. 17 used an independent adult cohort and distinct samples, images, molecular data, and cell experiments. The present *in vivo* analyses used a prespecified subset of the broader cohort reported in Ref. 18. Cortical immunohistochemistry was performed on the same brains and sections used previously for hippocampal analyses, but all cortical regions of interest, images, measurements, and numerical data are new. The cortical RT-qPCR measurements have not been published previously. The neonatal primary cultures were independent of both adult *in vivo* studies.

Table S1. Relationship between the present dataset and the authors' previous SDEA studies.

**Component**	**Ref. 17**	**Ref. 18**	**Present manuscript**
Adult *in vivo* cohort	Independent cohort. No shared animals or samples.	Same broader experimental cohort.	A prespecified subset of 11 mice per group: 7 for cortical immunohistochemistry and 4 for cortical RT-qPCR.
Histological material	No overlap.	Hippocampal regions and other hippocampal outcomes were reported previously.	New cortical regions of interest were analyzed on the same brains and sections. No previously published image or numerical value was reused.
Cortical RT-qPCR	No overlap.	Not reported.	New cortical measurements of *Gfap, C3, S100a10, Clcf1, Ppara, Pparg, Bdnf, Ngf*, and *Gdnf* (n = 4 mice per group).
Behavioral and other outcomes	Independent behavioral, microglial, inflammatory, and SIM-A9-cell outcomes.	Behavioral, hippocampal synaptic, dendritic-spine, astroglial, and proliferative outcomes were reported previously.	Previously published behavioral and hippocampal outcomes were not reanalyzed or presented.
Primary cultures	No overlap.	No overlap.	Independent neonatal astroglia-enriched cultures and new RT-qPCR data.

The overlap concerns the source cohort and, for immunohistochemistry, the underlying brain sections; the anatomical regions, images, measurements, and molecular endpoints reported here are new.
